# *ZNF385B* and *VEGFA* Are Strongly Differentially Expressed in Serous Ovarian Carcinomas and Correlate with Survival

**DOI:** 10.1371/journal.pone.0046317

**Published:** 2012-09-28

**Authors:** Bente Vilming Elgaaen, Ole Kristoffer Olstad, Leiv Sandvik, Elin Ødegaard, Torill Sauer, Anne Cathrine Staff, Kaare M. Gautvik

**Affiliations:** 1 Department of Gynaecological Oncology, Oslo University Hospital, Oslo, Norway; 2 Faculty of Medicine, University of Oslo, Oslo, Norway; 3 Department of Medical Biochemistry, Oslo University Hospital, Oslo, Norway; 4 Unit of Biostatistics and Epidemiology, Oslo University Hospital, Oslo, Norway; 5 Department of Pathology, Oslo University Hospital, Oslo, Norway; 6 Department of Gynaecology, Oslo University Hospital, Oslo, Norway; Barts & The London School of Medicine and Dentistry, Queen Mary University of London, United Kingdom

## Abstract

**Background:**

The oncogenesis of ovarian cancer is poorly understood. The aim of this study was to identify mRNAs differentially expressed between moderately and poorly differentiated (MD/PD) serous ovarian carcinomas (SC), serous ovarian borderline tumours (SBOT) and superficial scrapings from normal ovaries (SNO), and to correlate these mRNAs with clinical parameters including survival.

**Methods:**

Differences in mRNA expression between MD/PD SC, SBOT and SNO were analyzed by global gene expression profiling (n = 23), validated by RT-qPCR (n = 41) and correlated with clinical parameters.

**Results:**

Thirty mRNAs differentially expressed between MD/PD SC, SBOT and SNO were selected from the global gene expression analyses, and 21 were verified (p<0.01) by RT-qPCR. Of these, 13 mRNAs were differentially expressed in MD/PD SC compared with SNO (p<0.01) and were correlated with clinical parameters. ZNF385B was downregulated (FC = −130.5, p = 1.2×10^−7^) and correlated with overall survival (p = 0.03). VEGFA was upregulated (FC = 6.1, p = 6.0×10^−6^) and correlated with progression-free survival (p = 0.037). Increased levels of TPX2 and FOXM1 mRNAs (FC = 28.5, p = 2.7×10^−10^ and FC = 46.2, p = 5.6×10^−4^, respectively) correlated with normalization of CA125 (p = 0.03 and p = 0.044, respectively). Furthermore, we present a molecular pathway for MD/PD SC, including VEGFA, FOXM1, TPX2, BIRC5 and TOP2A, all significantly upregulated and directly interacting with TP53.

**Conclusions:**

We have identified 21 mRNAs differentially expressed (p<0.01) between MD/PD SC, SBOT and SNO. Thirteen were differentially expressed in MD/PD SC, including ZNF385B and VEGFA correlating with survival, and FOXM1 and TPX2 with normalization of CA125. We also present a molecular pathway for MD/PD SC.

## Introduction

Ovarian cancer is the fourth and fifth most frequent cause of cancer death in women in Norway and the United States, respectively [1], [2]. When diagnosed, about 65% of the patients have distant spread of disease (stage III–IV), and their 5-year relative survival rate is less than 30% [1], [2].

Epithelial ovarian cancer (EOC) constitutes more than 90% of ovarian cancers and comprises a heterogeneous group of tumours. Serous ovarian carcinomas (SC) are the most common histological subtype [3], [4], of which the moderately differentiated (MD) and poorly differentiated (PD) are predominant compared with the well differentiated (WD) [3]. It is generally understood that MD and PD SC represent a common tumour subclass distinct from that of WD SC and serous ovarian borderline tumours (SBOT) with respect to origin, pathogenesis, molecular profile and clinical outcome [5]–[11].

Several previous DNA microarray expression analyses of EOC have identified genes related to histology or clinical outcome parameters [12], [13]. A few DNA microarray expression analyses, restricted to the molecular differences between MD/PD SC and SBOT have been carried out [9], [10], [14], [15]. However, the differentially expressed mRNAs were not correlated with clinical parameters. Moreover, only one of these studies [9] included normal ovarian surface epithelium (OSE), which has been shown to be a valid control tissue [16].

Improved insight into the molecular characteristics of the different subgroups of EOC should eventually lead to more individualized and effective treatments. The aim of this study was to identify mRNAs differentially expressed between MD/PD SC, SBOT and superficial scrapings from normal ovaries (SNO), using global gene expression profiling and validation by RT-qPCR, and to correlate differentially expressed mRNAs of MD/PD SC with clinical parameters. In contrast to previous studies, we initially analyzed gene expression based on histological subgroups and then linked the differentially expressed mRNAs to clinical parameters. We have identified several subgroup characteristic mRNAs, including some with apparent clinical relevance.

## Materials and Methods

### Ethics Statement

The study was approved by the Regional Committee of Medical and Health Research Ethics (REK, ref.no. 530-02163) in Eastern Norway and all participants signed informed consent.

### Patients and Tissue Material

Women were recruited prior to operations for gynaecological diseases at Oslo University Hospital, Ulleval, in the period 2003 to 2007. Clinicopathological and laboratory information were obtained from hospital records and additional preoperative patient interviews. Tissue specimens were obtained from women previously not receiving chemotherapy, during their primary operation. SNO samples collected from patients operated for benign gynaecological diseases were used as control material [17]. By scraping the surface of normal ovaries gently with a scalpel, the vast majority of the harvested cells were verified cytologically as normal OSE cells, being positive for pankeratin by immunocytochemistry (data not shown). Immediately after harvesting the tissue samples were snap-frozen in liquid nitrogen, whereas the SNO samples were transferred to 500 µl TRIzol solution (Invitrogen.com) in order to avoid mRNA degradation. The samples were stored at −80°C until processed.

The histological classification and clinical stage were according to the World Health Organization classification of tumours and the International Federation of Gynecology and Obstetrics classification, respectively. The tumours were reviewed by two experienced and independent pathologists, and prior to RNA isolation a frozen section of all biopsies was examined to ensure satisfying sample quality and representativeness. By histological evaluation, only carcinomas presenting more than 50% tumour cells were included in the RT-qPCR analyses.

Global gene expression was carried out in eleven MD/PD SC, eight SBOT and four SNO samples. The tumours were selected and some pooled (n = 2–3) according to histological classification and stage, resulting in four MD/PD SC groups and four SBOT groups (Table 1). The SNO samples were analyzed individually. Differentially expressed candidate mRNAs were validated by RT-qPCR in all but three of these samples and in additional samples totalling 21 MD/PD SC, 13 SBOT and seven SNO, analyzed individually.

**Table 1:** Histological classification and group selection for patients selected for global gene expression analyses.

Group	Histological classification
1 (n = 3)	SC, MD, FIGO stage IIIC
2 (n = 3)	SC, PD, FIGO stage IIIC
3 (n = 3)	SC, 2MD, 1PD, FIGO stage IV
4 (n = 2)	SC, PD, FIGO stage IV
5 (n = 3)	SBOT, FIGO stage IA
6 (n = 1)	SBOT, FIGO stage IB
7 (n = 1)	SBOT, FIGO stage IC
8 (n = 3)	SBOT, FIGO stage II–III

SC: Serous ovarian carcinomas. MD: Moderately differentiated. PD: Poorly differentiated. SBOT: Serous ovarian borderline tumours. FIGO: International Federation of Gynecology and Obstetrics. A minor sarcoma component was retrospectively discovered in one SC, but was not found in the biopsy used, still excluded from RT-qPCR.

### RNA Preparation

Tissue specimens were homogenized directly for 2×2 minutes in 750 µl TRIzol using a Tissuelyzer (Qiagen.com). Total RNA was extracted using Trizol (Invitrogen, Carlsbad, CA) and further purified by the RNeasy MinElute cleanup kit (Qiagen catalog no. 74204) according to the manufacturer's instructions. The isolated total RNA was quantified (Nano Drop spectrophotometer; Saveen Werner AB) and quality controlled using the Agilent BioAnalyzer 2100 system and the RNA 6000 Nano assay. All samples showed high RNA quality.

### Global Gene Expression Profiling

Five micrograms of total RNA were used for analysis with the one-cycle cDNA synthesis kit following the manufacturer's (Affymetrix) recommended protocol for gene expression analysis. Biotinylated and fragmented cRNA was hybridized to the Affymetrix HG U133 Plus 2.0 array, representing 47000 transcripts for 38500 well characterized human genes. The signal intensities were detected with the Hewlett-Packard gene array scanner 3000 7G (Hewlett-Packard, Palo Alto, CA). Complete microarray expression data have been deposited in NCBI's Gene Expression Omnibus [18] (accession number GSE36668).

### Quantitative Reverse Transcription-Polymerase Chain Reaction (RT-qPCR)

RT-qPCR reactions were performed by using ABI Prism 7900 HT sequence detection system (Applied Biosystems). Microfluidic Taqman arrays were designed to measure the mRNA expression. Briefly, total RNA was reversely transcribed using Omniscript (Qiagen Ltd., Crawley, United Kingdom). 300 nanograms of cDNA were used per sample-loading port, each allowing 48 q-PCR reactions following the manufacturer's instructions. Each mRNA was run in triplicates. Based on high expression and negligible variation, the reference gene GAPDH was used to normalize gene expression levels.

Gene expression patterns were calculated using the comparative crossing threshold method of relative quantification (ΔΔCq method) [19], and presented as relative (ΔCq) and fold change (FC) values. ΔCq was designated as the mean quantification cycle (mean of triplicates) of an mRNA in a sample subtracted by the mean quantification cycle (mean of triplicates) of GAPDH in the same sample. ΔΔCq was calculated as mean ΔCq of the SNO subtracted by ΔCq of each tumour sample, whereas FC was 2^ΔΔCq^. ΔCq values were imported into Patrek Genomics Suite (Partek Inc., St Louis, MO, USA), and subjected to a non supervised cluster analysis using the euclidean/average linkage algorithm.

### Ingenuity Pathway Analysis

Ingenuity Pathway Analysis (Redwood City, CA) was used for classifying genes into biological functions and signalling pathways.

### Statistical Analyses

The eight groups classified in Table 1 and four samples of SNO were processed using GCOS 1.4 (Affymetrix). The CEL files were imported into Array Assist software (v5.2.0; Iobion Informatics LLC, La Jolla, CA) and normalized using the PLIER (probe logarithmic intensity error) algorithm in Array Assist to calculate relative signal values for each probe set. In order to filter for low signal values, the MAS5 algorithm in Array Assist was used to create a data set of absolute calls, showing the number of present and absent calls for each probe set. The filtration was performed by eliminating probe sets containing≥10 absent calls across the data set, resulting in a reduction of probe sets from 47000 to 32707. For expression comparisons of different groups, unpaired t-tests and Benjamini Hochberg correction of p-values for multiple testing were used.

When comparing ΔCq values in different histological subgroups, a two-sided independent sample t-test was used since the ΔCq values were close to normally distributed. Differentially expressed mRNAs given as FC values were correlated with clinical parameters. In order to decide whether an mRNA expression was significantly associated with time until death or time until progression, Cox regression analyses were used. When significant, Kaplan-Meier plots were used to estimate survival curves for tertiles of the expression variable. To compare mRNA expression levels in two groups of patients, a two-sided Mann-Whitney U-test was used, since the FC expression levels were not normally distributed. The results for each group are presented as medians. A significance level of 1% was used for differential mRNA expression, and 5% for correlating mRNAs with clinical parameters. The statistical analyses were performed by employing SPSS version 18.

## Results

### Patient Characteristics

Clinicopathological and laboratory information regarding patients selected for the RT-qPCR analyses is given in Table 2. The patients had no other diseases than ovarian cancer influencing survival, were in good preoperative condition [17] and were Caucasian except for one Latino SBOT patient. All were postmenopausal except for three SBOT patients, and no cancer patients were currently receiving hormone therapy. Primary debulking surgery was performed in all carcinoma patients, and with the exception of two patients, all received platinum-based adjuvant treatment.

**Table 2:** Clinicopathological and laboratory information for patients selected for RT-qPCR analyses.

Parameters	MD/PD SC^a^, n = 21	SBOT, n = 13
Age; mean ± SD (range)	69.0±9.9 (51–84)	58.5±14.9 (36–82)
Preoperative CA125 (kU/L); mean ± SD	3320±4761	350±714
FIGO stage		
I	n = 2 (IC)	n = 10 (6IA, 3IB, 1IC)
II	n = 1 (IIC)	n = 2 (1IIB, 1IIC)
III	n = 14 (1IIIB, 13IIIC)	n = 1 (IIIB)
IV	n = 4	n = 0
Residual tumour		
0 cm	n = 5	n = 12
<2 cm	n = 5	n = 1
>2 cm	n = 11	
Start of chemotherapy (days after surgery); mean ± SD	30.4±11.6	
CA125 response	n = 20	
Optimal CA125 normalization	n = 14	
Median time (months) until progression (95%CI)	13 (10–16)	
Median time (months) until death (95%CI)	29 (17–41)	
Status at last follow-up		
Alive, no EOC	n = 3	n = 12
Dead of EOC	n = 18	n = 0

a 12 MD, 9 PD. SD: Standard deviation. CI: Confidence Interval. Further abbreviations are given in Table 1.

Follow-up data (Table 2), including clinical examinations, standard laboratory analyses and complementary diagnostic imaging were available for all patients. The protein CA125 (cancer antigen 125) was measured prior to each chemotherapy cycle and was used as a marker for response to therapy. A CA125 response was defined according to The Gynecologic Cancer Intergroup (GCIG) criteria, including at least a 50% reduction in CA125 levels from a pre-treatment sample. A CA125 normalization was defined as optimal when normalized (<35 kU/L) within four cycles of chemotherapy. After completion of treatment, the patients were evaluated every third months for two years, every six months for the next three years, and thereafter once a year. Progression-free survival (PFS) and overall survival (OS) were defined as the time interval from the date of surgery to the date of first confirmed disease recurrence and to the date of death, respectively. Disease progression was based on an increase in the CA125 level according to the GCIG criteria and a verified clinical relapse, and the date of the first event was used. Clinical data was current as of 25 August 2011.

### Global Gene Expression Analyses and RT-qPCR Validation

From 47000 transcripts a comparison between MD/PD SC, SBOT and SNO was made to detect differentially expressed mRNAs. Based on p-values (<0.005), FC values (>10) and visual investigation of the microarray cluster analysis heatmap, 30 mRNAs (Table 3) were selected for RT-qPCR validation. The global mRNA expression results were largely confirmed by the RT-qPCR analyses. By applying a significance level of 1%, 21 of 30 mRNAs were verified as differentially expressed between MD/PD SC, SBOT and SNO (Table 4). Twenty of these mRNAs were markedly differentially expressed (p<0.005), including 14 with a p<10^−5^. Thirteen mRNAs distinguished MD/PD SC from SNO (ten up- and three down-regulated). ZNF385B was the most differentially expressed mRNA according to the FC value (p = 1.2×10^−7^, FC = −130.5), followed by LCN2, CRISP2 and FOXM1. When comparing MD/PD SC with SNO and SBOT, respectively, ten mRNAs were similarly differentially expressed. Eight mRNAs were differentially expressed in MD/PD SC only when compared with SBOT, including DNAH9 (p = 1.6×10^−10^, FC = −414.5). Comparison of SBOT and SNO showed an entirely different pattern (Table 4). When SC were subgrouped into MD and PD tumours and separately compared with SNO and SBOT (t-test of ΔCq values), similar profiles were found for the two subgroups (data not shown).

**Table 3:** Differentially expressed mRNAs selected for RT-qPCR validation.

Symbol	Title	Biological function
ALPP	Alkaline phosphatase, placental	Metabolism
BIRC5	Baculoviral IAP repeat containing 5	Cell proliferation
CRABP2	Cellular retinoic acid binding protein 2	Transcription
CRISP2	Cysteine-rich secretory protein 2	Cell-cell adhesion
CRISP3	Cysteine-rich secretory protein 3	Immune response
CTCFL	CCCTC-binding factor (zinc finger protein)-like	Transcription
DNAH9	Dynein, axonemal, heavy chain 9	Cell motility
DYNLRB2	Dynein, light chain, roadblock-type 2	Metabolism
FOXM1	Forkhead box M1	Transcription
GRIA2	Glutamate receptor, ionotropic, AMPA 2	Ion transport
HLA-DQB1	Major histocompatibility complex, class II, DQ beta 1	Immune response
HLA-DRB1	Major histocompatibility complex, class II, DR beta 1	Immune response
KLK8	Kallikrein-related peptidase 8	Proteolysis
LCN2	Lipocalin 2	Immune response
MMP10	Matrix metallopeptidase 10 (stromelysin 2)	Proteolysis
PRAME	Preferentially expressed antigen in melanoma	Transcription
PROM1	Prominin 1	Signal transduction
PTH2R	Parathyroid hormone 2 receptor	Signal transduction
RBFOX1	RNA binding protein, fox-1 homolog (C. elegans) 1	RNA processing
S100A8	S100 calcium binding protein A8	Inflammatory response
SCEL	Sciellin	Cell differentiation
SFRP2	Secreted frizzled-related protein 2	Cell differentiation
SST	Somatostatin	Signal transduction
TMEM190	Transmembrane protein 190	Unknown
TOP2A	Topoisomerase (DNA) II alpha 170 kDa	Transcription
TPPP3	Tubulin polymerization-promoting protein family member 3	Microtubule bundle formation
TPX2	Microtubule-associated, homolog (Xenopus laevis)	Cell proliferation
VEGFA	Vascular endothelial growth factor A	Cell proliferation, angiogenesis
ZIC1	Zic family member 1	Cell differentiation
ZNF385B	Zinc finger protein 385B	DNA binding

According to Ingenuity Systems.

**Table 4:** Differentially expressed mRNAs (p<0.01) between MD/PD SC, SBOT and SNO.

	MD/PD SC vs. SNO		MD/PD SC vs. SBOT		SBOT vs. SNO	
mRNAs	p-values	FC values	p-values	FC values	p-values	FC values
**ALPP**			4.3×10^−6^	−17.0	4.4×10^−4^	10.3
**BIRC5**	1.2×10^−3^	24.4	1.6×10^−10^	8.5		
**CRABP2**	2.6×10^−7^	20.4	8.8×10^−8^	10.9		
**CRISP2**	2.1×10^−3^	−57.9				
**CRISP3**			7.6×10^−4^	−60.4		
**CTCFL**	1.5×10^−3^	34.7	3.4×10^−6^	60.8		
**DNAH9**			1.6×10^−10^	−414.5	2.3×10^−5^	32.7
**DYNLRB2**	2.2×10^−3^	−6.9	1.0×10^−7^	−23.1	1.4×10^−3^	3.4
**FOXM1**	5.6×10^−4^	46.2	1.4×10^−10^	14.8		
**KLK8**	1.3×10^−3^	40.1			5.5×10^−5^	28.1
**LCN2**	2.2×10^−5^	113.7			2.0×10^−3^	170.4
**PTH2R**	8.0×10^−6^	41.3	4.3×10^−7^	50.3		
**RBFOX1**			2.9×10^−3^	−14.2		
**S100A8**			1.6×10^−3^	5.6		
**TMEM190**			4.1×10^−9^	−66.4	5.0×10^−7^	50.3
**TOP2A**	1.5×10^−9^	30.4	1.4×10^−6^	5.2	1.5×10^−4^	5.9
**TPPP3**			6.3×10^−11^	−21.9	2.1×10^−5^	7.8
**TPX2**	2.7×10^−10^	28.5	1.5×10^−13^	10.5	2.8×10^−3^	2.7
**VEGFA**	6.0×10^−6^	6.1	8.3×10^−6^	3.1		
**ZIC1**			8.6×10^−3^	11.3		
**ZNF385B**	1.2×10^−7^	−130.5	8.1×10^−4^	−11.4	6.6×10^−3^	−11.5

- illustrate downregulation. FC: Fold change. Further abbreviations are given in Table 1and 3.

**Figure 1:**
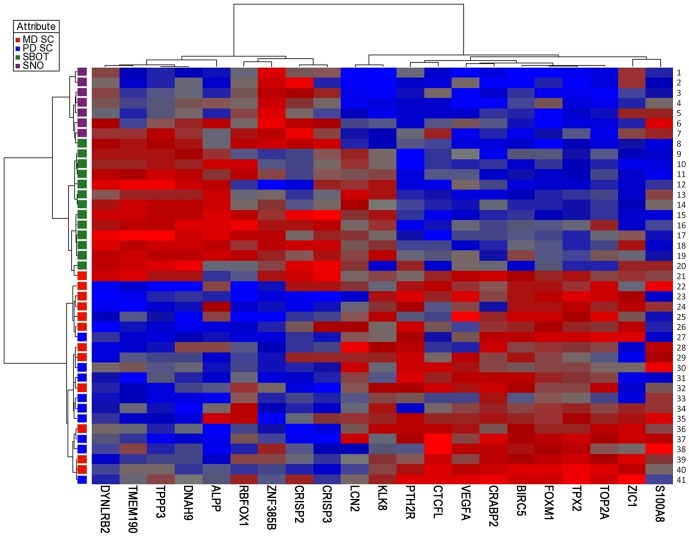
Cluster analysis heatmap. Cluster analysis heatmap of the expression levels (ΔCq values) of 21 differentially expressed mRNAs (p<0.01) in moderately (MD) and poorly differentiated (PD) serous ovarian carcinomas (SC), serous ovarian borderline tumours (SBOT) and superficial scrapings from normal ovaries (SNO). Each column represents an mRNA and each row a sample. The more over-and under-expressed the mRNA, the brighter the red and blue colour, respectively. Due to technical analysis errors for DYNLRB2, CRABP2, CRISP2, CRISP3 and LCN2 in sample nr 7, 21 and 26, these values are calculated as the mean ΔCq values of each subgroup. Further abbreviations are given in Table 3.

Figure 1 visualises a cluster analysis heatmap of the expression levels of the 21 differentially expressed mRNAs (p<0.01), showing that MD/PD SC, SBOT and SNO are almost perfectly segregated. Generally, the MD/PD SC mRNA expression levels clustered together as did those of SBOT and SNO. Two distinct portraits appeared, illustrating differential expression of these mRNAs in MD/PD SC versus both SBOT and SNO, whereas SBOT and SNO showed more similar patterns. The SC was separated into MD and PD, and their portraits overlapped considerably. Notably, the expression of BIRC5, FOXM1, TPX2 and TOP2A clustered together, adjacent to the cluster with VEGFA.

### Ingenuity Pathway Analysis

**Figure 2:**
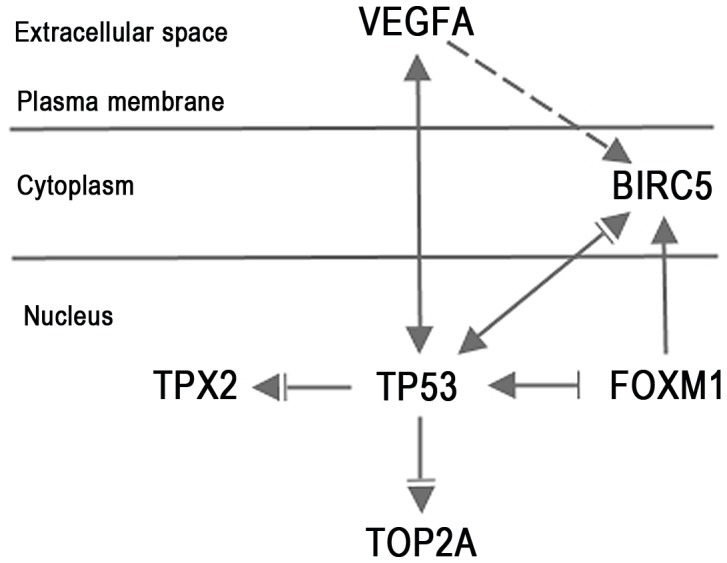
Molecular pathway for moderately and poorly differentiated serous ovarian carcinomas. ▾acts on (– direct interaction, -- indirect interaction), _⊥_ inhibits. The pathway was facilitated through Ingenuity Pathway Analysis. Abbreviations are given in Table 3.

The 21 differentially expressed mRNAs (Table 4) were mapped in the Ingenuity Pathways of Knowledge Base. Comparison of MD/PD SC with SNO revealed two connecting networks linked together by FOXM1. These two networks included all the 13 differentially expressed mRNAs in MD/PD SC. One of the networks (Fig. S1) included nine of the 13 mRNAs (BIRC5, CRABP2, DYNLRB2, FOXM1, KLK8, LCN2, TOP2A, TPX2 and VEGFA), whereas the other network (not shown) included five of the 13 mRNAs (CRISP2, CTCFL, FOXM1, PTH2R and ZNF385B). Direct interactions between five of the most significantly upregulated mRNAs shown in Figure S1 (VEGFA, FOXM1, TPX2, BIRC5 and TOP2A) and the tumour suppressor gene TP53 (tumour protein p53) were found, and a core pathway for MD/PD SC was generated (Fig. 2). In retrospect, the microarray analyses showed that TP53 was highly, although not among the most differentially expressed mRNAs in MD/PD SC compared with both SBOT (p = 2.5×10^−4^, FC = 2.4) and SNO (p = 1.2×10^−3^, FC = 2.0). The molecular interactions of the pathway were related to mRNAs, DNA and proteins.

### Correlation of mRNA Expression with Clinical Parameters

**Figure 3:**
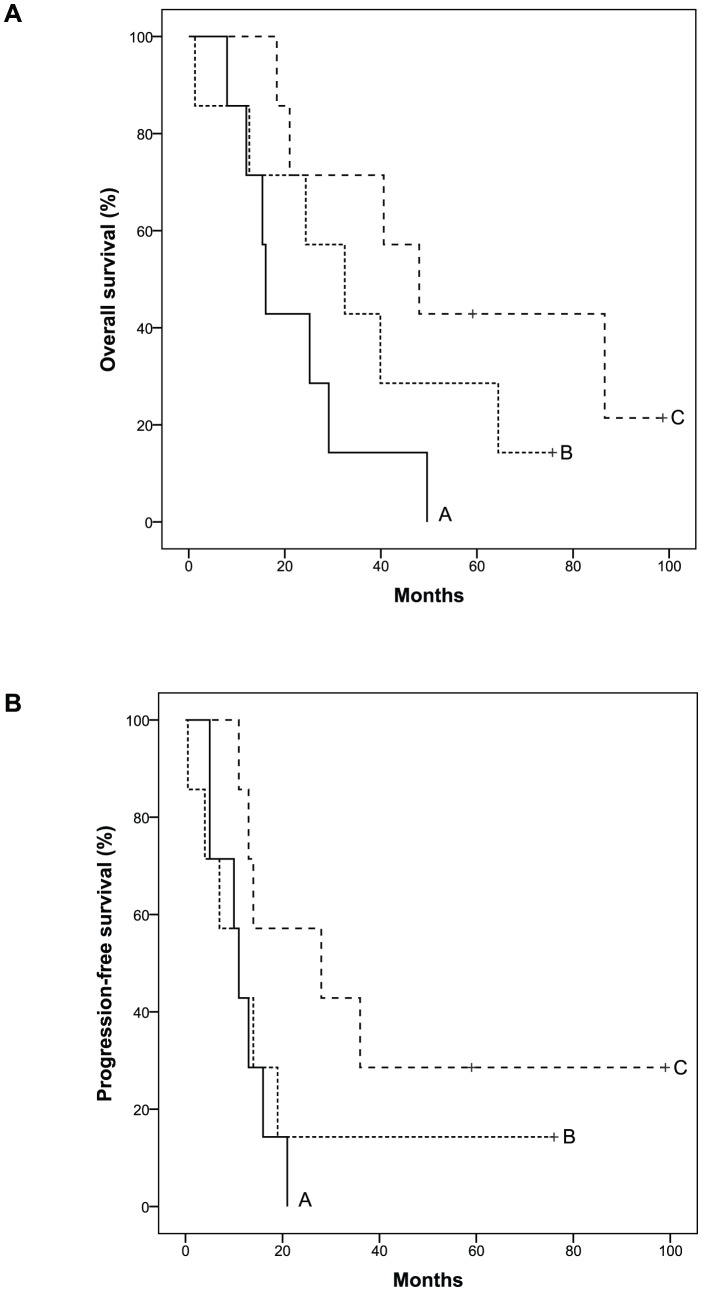
Kaplan-Meier survival curves. Overall survival curves according to ZNF385B mRNA expression level (FC) tertiles (**A**) and progression-free survival curves according to VEGFA mRNA expression level (FC) tertiles (**B**) in patients with moderately and poorly differentiated serous ovarian carcinomas. A: High expression. B: Intermediate expression. C: Low expression.

The 13 differentially expressed mRNAs in MD/PD SC compared with SNO (Table 4) were correlated with OS, PFS, optimal CA125 normalization after treatment and residual tumour amount after surgery. ZNF385B and VEGFA were associated with OS (p = 0.03) and PFS (p = 0.037), respectively. The ZNF385B and VEGFA expression levels for MD/PD SC were divided into tertiles, and Kaplan-Meier plots made (Fig. 3A–B). Patients with the lowest tertile of ZNF385B expression level had a much longer OS than patients with the highest tertile level, with median time until death of 48 and 16 months, respectively. In the intermediate ZNF385B tertile group the average median time until death was 32 months, averaging the survival times for the high and low ZNF385B tertile groups. Patients with the lowest VEGFA expression levels had a much longer PFS than patients with the highest and intermediate levels, with median time until progression of 28 and 11 months, respectively. When adjusting for FIGO stage, the associations between ZNF385B and OS as well as VEGFA and PFS were still significant (p = 0.030 and p = 0.031, respectively).

TPX2 and FOXM1 correlated with optimal CA125 normalization (p = 0.03 and p = 0.044, respectively). Patients with optimal CA125 normalization had higher expression levels of TPX2 and FOXM1 (n = 14; median FC = 33.0 and 51.2, respectively) than patients without optimal CA125 normalization (n = 7; median FC = 14.2 and 20.7, respectively). No association between the 13 differentially expressed mRNAs and residual tumour amount was found.

## Discussion

The mRNA profile of MD/PD SC was clearly different from that of SBOT and SNO, while the latter two showed marked similarities. In fact, the mRNAs differentially expressed in MD/PD SC showed predominantly inverse heatmap portraits compared with SBOT/SNO. A lower potential of malignancy combined with a reduced proportion of tumour cells in SBOT compared with MD/PD SC may at least partly explain the similar gene expression in SBOT and SNO. The similar mRNA expression profiles of MD and PD SC have been recognized previously [10].

Expression of ZNF385B was 130 times less in MD/PD SC compared with SNO, and the degree of downregulation correlated positively with OS. ZNF385B belongs to the family of zinc-finger genes, which encode transcription factors, playing an essential role in gene expression. This mRNA is supposed to be a transcription repressor, but the specific target genes have not been identified [20]. We hypothesize that its repression of transcription somehow inhibits neoplasia and/or tumour cell metastasis. Thus, when the transcriptional inhibition of ZNF385B decreases and mRNA levels increase, tumour growth/metastasis is promoted, resulting in shorter OS. The present study is to our knowledge the first to link ZNF385B to ovarian cancer.

VEGFA, a major mediator of tumour angiogenesis [21], was significantly upregulated in MD/PD SC, and a high expression was associated with a short progression-free survival. Consistent with our findings, a high expression of VEGFA as well as an association with poor prognosis have previously been found in malignant tumours including EOC [22]–[25], indicating that VEGFA may be a possible prognostic marker. A humanized monoclonal antibody targeting VEGFA, Bevacizumab, has been approved for the treatment of several tumour types, including EOC [21], [26], [27]. For ovarian cancer patients Bevacizumab in combination with standard chemotherapy has shown improvement in PFS in several phase III trials, including front line [26], [27] and platinum-resistant recurrent [28] treatment.

High expression levels of TPX2 and FOXM1 correlated with optimal CA125 normalization, and were among the most markedly upregulated mRNAs in MD/PD SC compared with both SNO and SBOT. Thus, effective chemotherapy appears to be associated with upregulation of these genes.

TPX2 has an important function in spindle assembly during cell division [29] and has previously been shown to be overexpressed in ovarian cancer, including MD/PD SC, and other malignancies [10], [30]–[32]. TPX2 is an activator of AURKA (aurora kinase A) [33], [34], which is overexpressed in cancer and regarded as a key regulator of mitosis [33]. There is an overexpression of both AURKA and TPX2 in many different cancer forms, including ovarian cancer [24], [33], and it has been proposed that TPX2 and AURKA is a functional unit with oncogenic properties [33]. In concordance we found that AURKA was upregulated in MD/PD SC compared with SNO (p = 0.10, FC = 3.8) and SBOT (p = 5.9×10^−4^, FC = 6.3).

FOXM1 encodes a transcriptional activator involved in cell proliferation, and is overexpressed in various human malignancies, including ovarian carcinomas [35], [36]. FOXM1 promotes metastasis [37], and correlates with poor prognosis [36]. FOXM1 regulates several genes involved in the cell cycle progression, including BIRC5 and TP53 and is regulated by TP53 [38], [39]. TP53 represses FOXM1 after DNA damage [39], and the high rate of TP53 mutation in MD/PD SC has therefore been suggested to contribute to FOXM1 overexpression [11], in support of our presented MD/PD SC pathway.

Overexpression of FOXM1 [11], [14] and BIRC5 [11], [14], [40] in MD/PD SC has previously been described, also when compared with SBOT [14], strengthening the relevance of the present results. Also, a FOXM1 transcription factor network, including BIRC5 has recently been identified for MD/PD SC, in support of our findings [11]. BIRC5, also repressed by TP53 protein [41], [42], encodes survivin, which is regarded as one of the most cancer specific proteins identified, inhibiting apoptosis and promoting cell proliferation [41]–[43]. Survivin is expressed in about 90% of EOC, and appears to be a prognostic marker [44], [45]. Strategies for inhibiting BIRC5 are now utilized in several ongoing clinical trials on different cancer forms [43], but so far not in ovarian cancer. Our results suggest that BIRC5 might be a potential target for therapy in EOC.

A molecular pathway for MD/PD SC was identified, involving five markedly upregulated mRNAs (VEGFA, FOXM1, TPX2, BIRC5 and TOP2A), all directly interacting with TP53. The fact that TP53 was upregulated in MD/PD SC may represent a compensatory mechanism, since TP53 is mutated in almost all MD/PD SC [11], resulting in high levels of dysfunctional proteins. A normal TP53 protein inhibits all mRNAs in the pathway, but VEGFA. We postulate that a mutation in the TP53 gene results in a decreased inhibition and consequently an upregulation of FOXM1, TPX2, BIRC5 and TOP2A.

### Conclusions

We have identified several known and hitherto partly unrecognized mRNAs as significantly differentially expressed between MD/PD SC, SBOT and SNO, including a set with apparent clinical relevance. In spite of the relatively small sample size, we have found several significant associations between mortality/morbidity and gene expressions in patients with MD/PD SC. Survival curves indicate that these associations are strong and of clinical importance. ZNF385B, previously unrecognized as a potential ovarian tumour marker, and VEGFA correlated with overall and progression-free survival, respectively, whereas TPX2 and FOXM1 with optimal CA125 normalization. However, the novel findings should be interpreted with caution until verified in larger studies. We also present a molecular pathway facilitated through Ingenuity Pathway Analysis for MD/PD SC, including VEGFA, FOXM1, TPX2, BIRC5 and TOP2A, all directly interacting with TP53, possibly representing a carcinogenic hierarchical molecular structure. Mechanistic studies will be needed to test the functional associations postulated in this pathway in MD/PD SC. The identified mRNAs should be explored in future studies as candidates for potential biomarkers and targets for therapy.

## Supporting Information

Figure S1 **Network of molecular interactions for moderately and poorly differentiated serous ovarian carcinomas.** ▾acts on (– direct interaction, -- indirect interaction), _⊥_ inhibits. The network was generated by Ingenuity Pathway Analysis.
(TIF)
