# Supplementary figures and images for: ZNF385B and VEGFA Are Strongly Differentially Expressed in Serous Ovarian Carcinomas and Correlate with Survival
Source: PLoS One. 2012 Sep 28;7(9):e46317. doi: 10.1371/journal.pone.0046317 (PMC3460818; doi:10.1371/journal.pone.0046317)

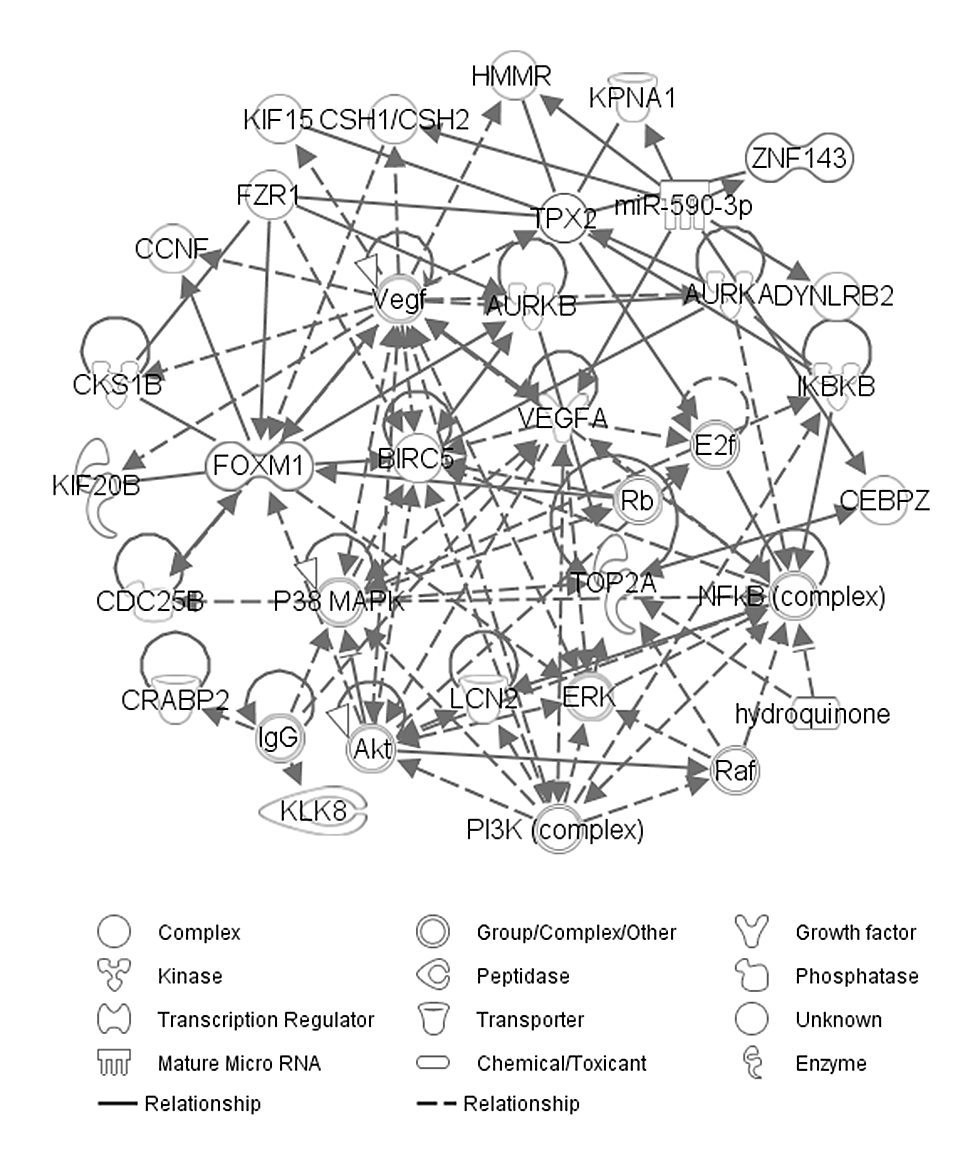

Supplement: Figure S1 — Network of molecular interactions for moderately and poorly differentiated serous ovarian carcinomas. ▾acts on (– direct interaction, -- indirect interaction), ⊥ inhibits. The network was generated by Ingenuity Pathway Analysis. (TIF) [file pone.0046317.s001.tif]
